# Reduced cell cohesiveness of outgrowths from eccrine sweat glands delays wound closure in elderly skin

**DOI:** 10.1111/acel.12493

**Published:** 2016-05-17

**Authors:** Laure Rittié, Elyssa A. Farr, Jeffrey S. Orringer, John J. Voorhees, Gary J. Fisher

**Affiliations:** ^1^ Department of DermatologyUniversity of Michigan Medical SchoolAnn ArborMIUSA

**Keywords:** aging, cell–cell contact, eccrine sweat gland, extracellular matrix, mechanical forces, skin, wound healing

## Abstract

Human skin heals more slowly in aged vs. young adults, but the mechanism for this delay is unclear. In humans, eccrine sweat glands (ESGs) and hair follicles underlying wounds generate cohesive keratinocyte outgrowths that expand to form the new epidermis. Here, we compared the re‐epithelialization of partial‐thickness wounds created on the forearm of healthy young (< 40 yo) and aged (> 70 yo) adults. Our results confirm that the outgrowth of cells from ESGs is a major feature of repair in young skin. Strikingly, in aged skin, although ESG density is unaltered, less than 50% of the ESGs generate epithelial outgrowths during repair (vs. 100% in young). Surprisingly, aging does not alter the wound‐induced proliferation response in hair follicles or ESGs. Instead, there is an overall reduced cohesiveness of keratinocytes in aged skin. Reduced cell–cell cohesiveness was most obvious in ESG‐derived outgrowths that, when present, were surrounded by unconnected cells in the scab overlaying aged wounds. Reduced cell–cell contact persisted during the repair process, with increased intercellular spacing and reduced number of desmosomes. Together, reduced outgrowths of ESG (i) reduce the initial number of cells participating in epidermal repair, (ii) delay wound closure, and (iii) lead to a thinner repaired epidermis in aged vs. young skin. Failure to form cohesive ESG outgrowths may reflect impaired interactions of keratinocytes with the damaged ECM in aged skin. Our findings provide a framework to better understand the mediators of delayed re‐epithelialization in aging and further support the importance of ESGs for the repair of human wounds.

## Introduction

Healing of skin wounds occurs *via* the succession of overlapping inflammatory, proliferative, and remodeling phases to, respectively, cleanse, close, and remodel the wound site. Overall, this complex biological process is aimed at restoring barrier function and physical properties of the skin. Many factors can hamper healing, including comorbid conditions (diabetes, venous insufficiency, immune suppression), wound factors (infection, unrelieved pressure at the surface of the wound), or physiological factors (nutrition, age). Although patients with nonhealing wounds often present with a combination of two or more of the above aggravating factors, many nonhealing wounds develop in the elderly (Allman, 1997; Jaul, 2009).

While the observation of the relationship between age and speed of healing was first reported a century ago (Du Noüy, 1916b), the mechanistic basis for this observation remains elusive. Reasons for this lack of understanding include variability of animal models studied, imprecise definition of aging, complexity of the wound healing process, and lack of control for comorbidities [reviewed in (Eaglstein, 1989; Sen *et al*., 2009; Sgonc & Gruber, 2013; Kim *et al*., 2015)]. Preferably, the effects of age on wound healing would be delineated in humans, in controlled conditions (i.e., similar wounds, wound sites, and wound care), and in the absence of comorbidities.

To circumvent inherent difficulties of studying natural skin wounds in humans, we developed a human wound healing model that utilizes a CO_2_ laser to generate partial‐thickness wounds. Wounds created by this procedure are highly reproducible and heal according to a typical repair process that can be readily studied (Orringer *et al*., 2004; Rittié *et al*., 2011, 2013). Using this method, we have shown that humans heal in a unique way, in that eccrine sweat glands (ESGs) play a major role in contributing new keratinocytes for epidermal repair, alongside less abundant hair follicles (Rittié *et al*., 2013).

ESGs are present in the paws of most mammals and are evenly distributed throughout the body skin of humans and a few primates. While paw/palm/sole ESGs produce sweat to increase adherence and grip, body ESGs fulfill the vital function of thermoregulation (Sato & Dobson, 1970) and produce sweat that maintains an acidic pH at the surface of the skin to limit commensal growth (Marples, 1965; Weller *et al*., 1996). Aging reduces all of the above‐mentioned sweat gland functions [reviewed in (Rittié & Fisher, 2015)] and alters the wound healing process (Du Noüy, 1916b; Holt *et al*., 1992; Gerstein *et al*., 1993; Thomas, 2001; Gosain & DiPietro, 2004; Sgonc & Gruber, 2013). Therefore, we sought to investigate the effects of aging on the re‐epithelialization function of ESGs. Our results confirm that the outgrowth of cells from ESGs is a major feature of repair in young skin. In addition, we show that less than 50% of the ESGs contribute to epidermal re‐epithelialization in aged skin. Surprisingly, we observe that the reduced number of epithelial outgrowths does not result from altered ESG number or proliferation in response to wounding, but rather from reduced cohesiveness of ESG progeny during repair.

## Results

### Reduced outgrowth of ESGs delays wound closure in aged individuals

**Figure 1:**
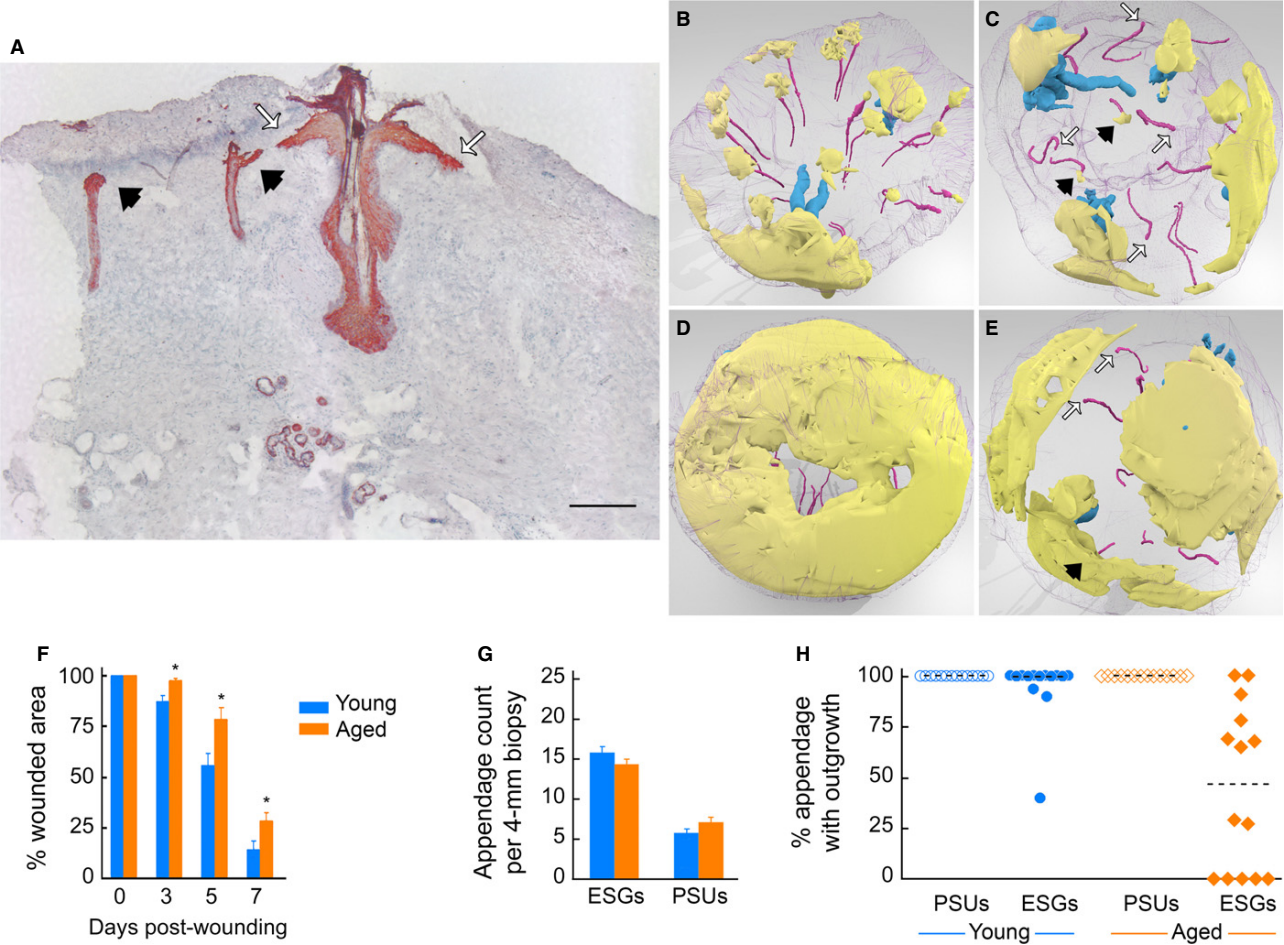
Reduced contribution of ESGs delays wound closure in aged individuals. (A) Pan‐keratin staining of transversally cut skin section obtained 3 days post‐wounding. Outgrowths from eccrine sweat glands (ESGs, black arrowheads) and pilo‐sebaceous unit (PSUs, white arrows) expand between the scab and the dermis. Positive staining is red, and hematoxylin counterstaining is blue. Scale bar = 250 μm. (B–E) Still images of 3D reconstructions (top views) of representative young (B, D) and aged (C, E) wounded human skin samples, taken at 5 (B, C) and 7 (D, E) days post‐wounding. Video S1 contains the corresponding animated versions. (B) In young human skin, ESGs (magenta, colors are arbitrary) and PSUs (blue) give rise to individual keratinocyte outgrowths (yellow) 5 days post‐wounding. Purple mesh is biopsy sample contours. (C) In aged skin 5 days post‐wounding, epidermal outgrowths are readily observed above PSUs and some ESGs (black arrows), while a subset of ESGs do not produce visible outgrowth (white arrows). (D) Seven days post‐wounding, young skin samples are nearly fully re‐epithelialized. (E) Seven days post‐wounding, aged wounded samples show comparatively delayed re‐epithelialization and lack of participation of a subset of ESGs (white arrows). (F) Re‐epithelialization is delayed in aged vs. young skin: Wound area coverage was measured over time in young (*n* = 5/time point) and aged (*n* = 5–9/time point). *: *P* < 0.05 vs. young. (G) ESGs and PSUs count in young (*n* = 20) and aged (*n* = 12) 4‐mm forearm biopsies. Differences in ESGs, PSUs or total appendage count (i.e., ESGs + PSUs, not depicted) were not statistically significant between age groups. (H) Percent ESGs with outgrowth in young (blue) and aged (orange) individuals. Each dot represents an individual; dotted lines are median values; difference between groups is statistically significant (*P* = 0.0005; *n* = 14/age group).

Our prior studies showed that, under our experimental conditions, CO_2_ laser treatment ablated the entire epidermis, the basement membrane, and the uppermost portion of the papillary dermis (Rittié *et al*., 2011, 2013). Moreover, we showed that keratinocyte outgrowths from ESGs are major contributors to re‐epithelialization of partial‐thickness wounds in young adults. Here, we asked whether ESG re‐epithelialization function was affected by aging. To this end, we compared skin re‐epithelialization after CO_2_ laser wounding in forearm skin samples taken from young (< 40 years) and aged (> 70 years) healthy individuals. Consecutive horizontally cut skin sections were immunostained for epithelial markers (pan‐keratin) and photographed, and the images were reassembled to create 3D reconstructions of skin, using computer software as described in Experimental Procedures. As previously reported (Rittié *et al*., 2013), we observed that young human skin produces discernible keratinocyte outgrowths from both ESGs and pilo‐sebaceous units (PSUs, i.e., hair follicles and associated sebaceous glands), within 3 days following wounding (a representative pan‐keratin‐stained transverse section is shown in Fig. 1A). Epidermal repair occurs through expansion of keratinocyte outgrowths merging with each other until they cover the entire wounded area ((Rittié *et al*., 2013) and Fig. 1; animated versions in Video S1). Re‐epithelialization is nearly complete 7 days post‐wounding in the young group (86 ± 4% of the wound re‐epithelialized). In the aged group, however, many ESGs had no discernible outgrowths, although all of the PSUs contributed outgrowths upon wounding (Fig. 1C). Lack of outgrowths from ESGs in aged skin was still observed 7 days post‐wounding (Fig. 1D,E), indicating that the ESG response was not simply deferred. We reasoned that as outgrowths expand laterally until they merge with one another, reduction in total number of outgrowths observed in elderly individuals should increase the time required for the merger to complete. Indeed, re‐epithelialization was delayed in the aged group, in which 72 ± 4% of the wound was re‐epithelialized 7 days post‐wounding (*n* = 9, roughly twice the remaining wounded area of the young group, that is, 28 ± 4% vs. 14 ± 4%, respectively, *P* = 0.022) (Fig. 1F).

We counted 15.8 ± 0.8 ESGs and 5.7 ± 0.5 PSUs per 4‐mm‐diameter forearm skin sample of young individuals, vs. 14.3 ± 0.6 ESGs and 7.1 ± 0.6 PSUs per 4‐mm‐diameter aged skin sample (Fig. 1G). The numbers of ESGs or PSUs in young vs. aged skin were not significantly different (*P* = 0.144, *P* = 0.093, respectively). Also, duct diameter was similar in young and aged skin and was not altered by wounding (not shown).

Collectively, we found that 13 of 14 young individuals (93%) had outgrowths from > 90% of their ESGs. In contrast, in 14 aged individuals only 3 (21%) had outgrowths from > 90% of ESGs, 6 (43%) had outgrowths from some ESGs, and 5 (36%) had no discernible ESG‐derived outgrowths. In all, median value for ESGs with outgrowths was 100% in the young group, vs. 47% in the aged group (*P* = 0.0005, Fig. 1H). In contrast, outgrowths were detected above 100% of PSUs, both in young and aged individuals (*n* = 14/group, Fig. 1H). Thus, delayed re‐epithelialization of partial‐thickness wounds in aged forearm skin *in vivo* is associated with reduced number of ESG with outgrowths, compared with young skin.

### Aging does not alter proliferative responses of PSUs or ESGs upon wounding

**Figure 2:**
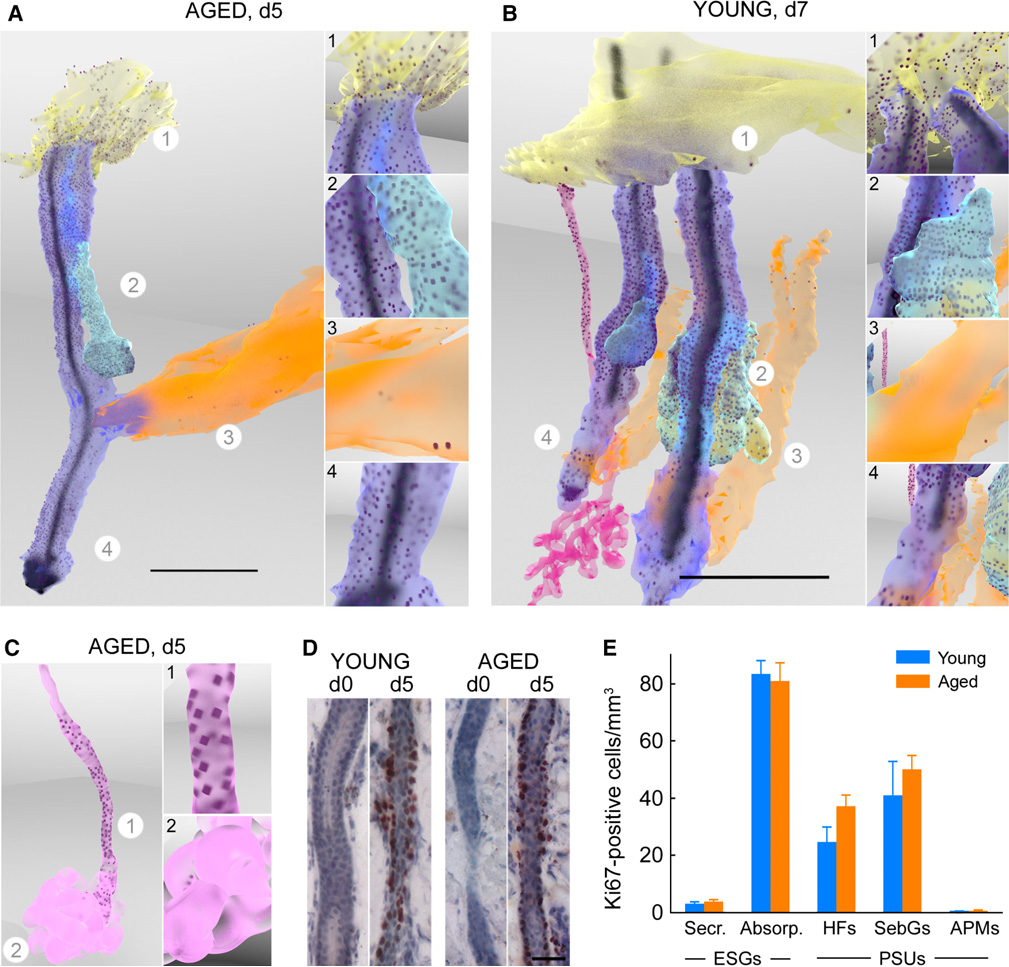
Aging does not alter proliferative response of PSUs and ESGs upon wounding. (A, B, C) Still images of 3D reconstructions of PSU (A, B) and ESG (B, C) in young (B) and aged (A, C) wounded human skin samples, taken 5 (A, C) and 7 (B) days post‐wounding. These particular examples are representative of young and aged samples, which harbor similar Ki‐67 distribution. Revolving 3D animated versions with close‐ups are provided as Video S2. (A, B) Insets on the right are single‐frame images of animated versions showing close‐ups of the areas indicated with a circled number on overview image. Scales bar = 500 μm. In PSUs (A, B), Ki‐67‐positive nuclei (dark dots) are located in virtually all of the cells of the outer root sheath of the hair follicle (purple), nearly all of the cells of the outer layer of the sebaceous glands (cyan), and most basal keratinocytes of the regenerative epidermis (yellow). A few scattered cells of the arrector pili muscle (orange) stain for Ki‐67. In ESGs (B, C), Ki‐67 highlights virtually all of the cells in the outer layer of the absorption portion (duct and proximal coil), and a few scattered cells in the secretory coils (most visible in C). (D) Ki‐67 staining of transversally cut skin sections showing similar proliferation in ESG ducts of wounded young and aged skin, but not unwounded samples. Positive staining is dark red, and hematoxylin counterstaining is blue. Scale bar = 50 μm. (E) Number of Ki67‐positive cells among appendage subunits 5 days post‐wounding normalized to respective subunit volume. Proliferation was similar between young and aged wounded samples. Ki67‐positive cells were relatively abundant in the absorption (Absorp.) portion of the eccrine sweat glands (ESGs), hair follicles (HFs), and sebaceous glands (SebGs), and relatively scarce in the secretory (Secr.) portion of the ESGs and in the arrector pili muscles (APMs). PSU: pilo‐sebaceous unit.

We previously reported that partial‐thickness wounding of young human skin triggers a proliferative response in ESGs and PSUs underlying the wound (Rittié *et al*., 2013). To assess whether reduced outgrowth from ESGs in aged skin stemmed from reduced proliferative response, we compared spatial distribution of proliferative cells within PSUs and ESGs in young and aged skins following wounding. Proliferative cells were identified by immunostaining with Ki‐67, a well‐accepted marker of proliferation (Bruno & Darzynkiewicz, 1992), and computer‐assisted 3D reconstructions of IHC images assembled as above. Compared with basal proliferation in unwounded skin, wounding triggers a strong proliferative response in ESGs and PSUs of young skin, as expected (Rittié *et al*., 2013). Five days postwounding, in PSUs underlying the wound, almost all of the cells of the outer root sheath (the outermost cell layer that wraps the entire hair follicle) and the vast majority of the outer layer of the sebaceous glands were positive for Ki‐67. The wound‐induced PSU proliferation response did not extend to the arrector pili muscle, which retained a relatively low proliferation level, similar to that in unwounded skin (Fig. 2A,B and animated versions with close‐ups in Video S2). The few hair follicles we saw in telogen (resting) phase had similar Ki67 distribution as their neighbor anagen (growing) hair follicles in the same skin sample (one example shown in Fig. 2B). At 7 days post‐wounding, proliferation appeared reduced in the outer root sheath located between the insertion of the arrector pili muscle and the sebaceous gland [the isthmus region, also designated as the bulge area in humans (Ohyama *et al*., 2006; Rittié *et al*., 2009)] (Fig. 2B and animated movies in supplementary data). Proliferation remained strong in other areas of the hair follicle, in sebaceous glands, and in the two‐most basal layers of the outgrowths (Fig. 2B).

Cell proliferation in ESGs was nearly undetectable in unwounded young skin, but was strongly induced in ESGs ducts by wounding, as previously reported (Lobitz *et al*., 1954; Rittié *et al*., 2013). Here, detailed observations indicated that virtually all of the cells in the outermost layer of ESG absorption portion (i.e., straight duct and proximal portion of the coil) were proliferative in response to wounding. In contrast, proliferation was minimal in the secretory portion of the ESGs (i.e., the majority of the coiled portion) (Fig. 2B,C, animated versions in Video S2).

Unexpectedly, we observed a very similar proliferative response following wounding in aged skin, both in ESGs and PSUs (examples in Fig. 2C,D, quantifications in Fig. 2E). These surprising data indicate that reduced ESG‐derived outgrowths in aged wounded skin do not result from lack of proliferative response.

### Aging is associated with reduced cohesiveness of ESG‐derived progeny

**Figure 3:**
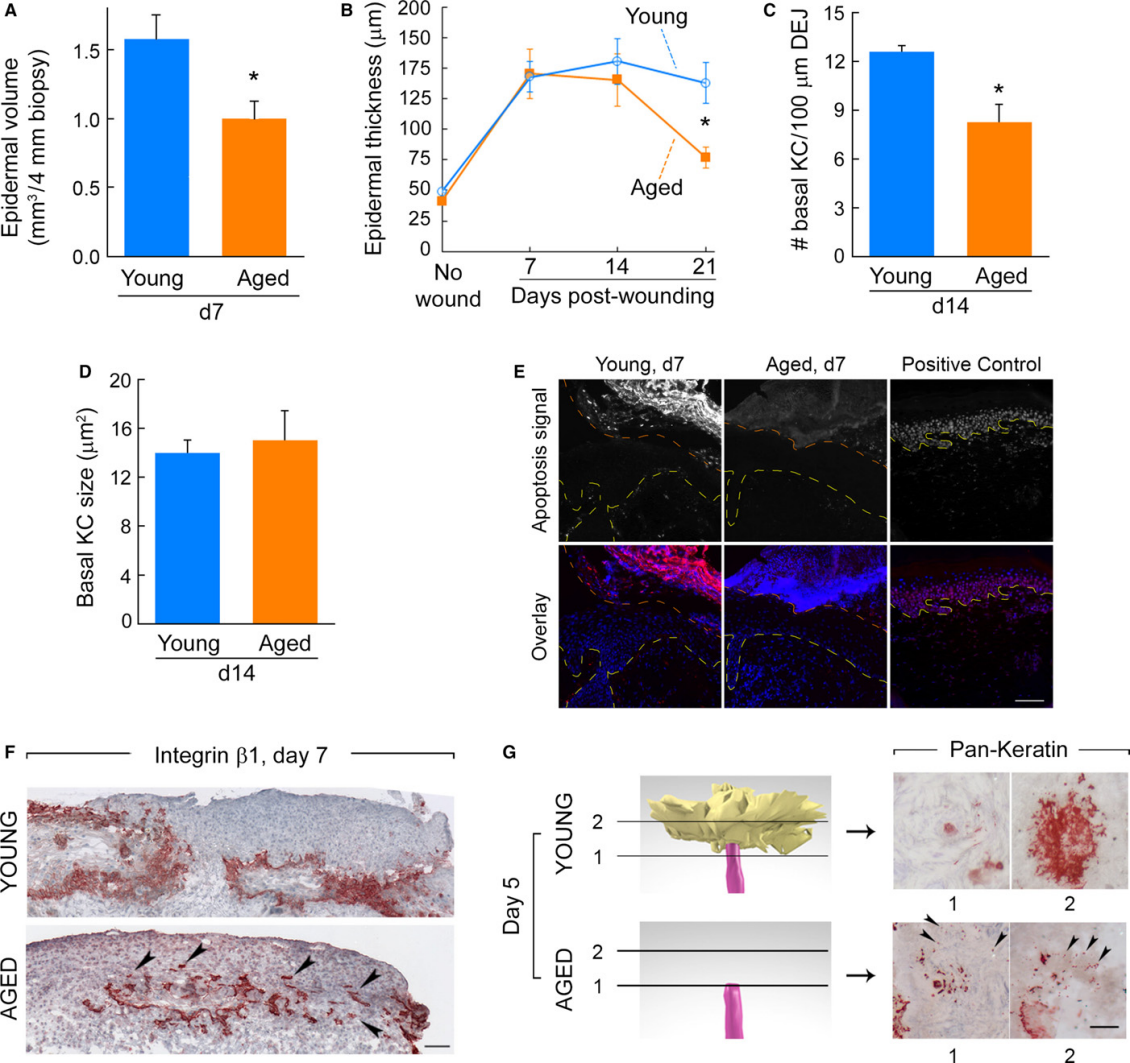
Aging is associated with reduced cohesiveness of ESG‐derived progeny. (A) Volume of repaired epidermis per 4‐mm biopsies in young and aged skins taken 7 days post‐wounding. (B) Epidermal thickness measured on 2D images in young and aged skin samples overtime. *N* = 7–9/age group for each time point. For all, *: *P* < 0.05 between young and aged. (C) Basal keratinocyte (KC) density. Number of KCs forming the basal layer was normalized to linear length of dermal–epidermal junction (DEJ). *N* = 6/age group. (D) Surface area of individual basal KCs. *N* = 6/age group. (E) Repaired epidermis is devoid of apoptotic cells in young and aged skin. DNA fragmentation was labeled with rhodamine (red fluorescence) using an apoptosis detection kit on wounded skin taken 7 days post‐wounding from young (left) and aged (middle) individuals. Positive control (right) is nonwounded skin digested with DNaseI. Top row: rhodamine fluorescence in grayscale (‘apoptosis signal’). Bottom row: overlay of apoptosis signal (red) and DAPI nuclear counterstaining (blue). Yellow lines delineate dermal–epidermal junctions; orange lines delineate epidermis–scab junction. Epidermis representative of 5 per age group. Scale bar = 100 μm. (F) Beta1‐integrin staining of transversally cut skin sections obtained 7 days post‐wounding. Positive staining is red, and hematoxylin counterstaining is blue. ESG‐derived outgrowths expand between the scab and the dermis in young (top) and aged (bottom) individuals (those with ESG‐derived outgrowths). Arrows in bottom panel point to isolated groups of cells present mostly in the scab and without apparent cohesion with the rest of the ESG‐derived outgrowth. Scale bar = 100 μm. (G) *Left*: 3D reconstruction of skin sample obtained 5 days post‐wounding showing a close‐up of the topmost portion of a representative ESG duct in young (top, with outgrowth) or aged (bottom, no outgrowth). Arbitrary colors are magenta for ESGs and yellow for outgrowth. *Right*: pan‐keratin‐immunostained horizontal sections used to make the 3D reconstructions; shown are two sections corresponding to the cutting planes (1 and 2) represented on 3D reconstruction in left panels. In young samples (top), pan‐keratin‐positive cells (red) form cohesive outgrowths above eccrine ducts. In aged samples, numerous pan‐keratin‐positive isolated cells surround the duct of ESGs (arrows), but do not form cohesive outgrowths. Scale bar = 50 μm.

With reduced ESG contribution but similar PSU involvement, the volume of repaired epidermis in aged skin was 37% less than young skin 7 days post‐wounding (1.00 ± 0.13 and 1.58 ± 0.17 mm^3^ in aged vs. young, respectively, *P* = 0.030, *n* = 5/group) (Fig. 3A). However, when epidermis was present on vertical sections (i.e., in the vicinity of PSUs), epidermal thickness was similar between young and aged repaired skin 7 days post‐wounding (Fig. 3B). These results are consistent with similar proliferation and contribution to epidermal repair of PSUs in the two age groups. However, while epidermal thickness peaked 14 days postwounding in young individuals and remained relatively high 21 days post‐wounding, epidermal thickness peaked at 7 days post‐wounding in the aged group and sharply decreased thereafter. Indeed, the repaired epidermis averaged 137.62 ± 16.68 μm thickness in young individuals 3 weeks post‐wounding, but was 44% thinner in aged subjects (76.95 ± 8.60 μm, N = 6 per group, *P* = 0.013) (Fig. 3B). Interestingly, we found a 35% reduction in the basal keratinocyte density (cell number per length of dermal–epidermal junction) in aged vs. young repaired epidermis 2 weeks post‐wounding (12.63 ± 0.39 vs. 8.27 ± 1.07 cells/100‐μm‐length in young vs. aged, respectively, *P* = 0.012) (Fig. 3C). However, the mean cell size was similar between the two age groups (14.0 ± 1.0 vs. 15.0 ± 2.4 μm^2^ in young vs. aged, respectively, *P* = 0.711) (Fig. 3D). Taken together, these data suggest that the reduced overall epidermal thickness observed in aged repaired epidermis might be due to a reduced initial number of keratinocytes relative to young skin.

During the later stages of wound repair, decreased cellularity is often achieved by programmed cell death, through apoptosis of certain cell types (Desmoulière *et al*., 1995; Arya *et al*., 2014). Normally, keratinocytes that form new epidermis during wound healing do not undergo apoptosis. Nevertheless, we considered whether keratinocyte apoptosis could account for decreased outgrowth from ESGs in aged skin. We thus assessed a potential increase in apoptotic keratinocytes during aged skin repair *in situ via* specific labeling of nuclear DNA fragmentation (Gavrieli *et al*., 1992). We detected apoptotic cells in the scab of young and aged wounded samples as expected (apoptosis may appear different in these young and aged scabs but we did not collect enough samples with intact scabs to ascertain this possibility). However, we did not find any apoptotic keratinocytes in the repairing epidermis of young or aged skin 1 week (Fig. 3E, representative of *N* = 5/age group) or 2 weeks (not shown) post‐wounding. These results ruled out that reduced initial number of keratinocytes in aged repaired epidermis was due to apoptosis.

We next compared the distribution of PSU and ESG‐derived progeny between young and aged skins using β1‐integrin, a cell surface receptor typically expressed by hyperproliferative keratinocytes (Lopez‐Rovira *et al*., 2005; Ernst *et al*., 2013) and crucial for keratinocyte migration and wound re‐epithelialization in mice (Grose *et al*., 2002). Results from these experiments revealed individual groups of cells disconnected from the larger outgrowths, in the scabs of the few aged individuals in whom a subset of ESGs gave rise to outgrowths. These groups of disconnected cells were not found in young skin (Fig. 3F). These observations suggested that, in aged skin, a significant proportion of proliferating keratinocytes, possibly those arising from the ESG, are not incorporated in (or detach from) cohesive outgrowths, and get trapped in the scab.

Having observed robust cell proliferation in ESGs in aged skin, we considered the possibility that failure to form discernible outgrowths resulted from reduced cohesiveness of ESG cells at the top of the ducts. Therefore, we re‐examined the pan‐keratin‐stained horizontal sections at high magnification (400×). We did not observe any thickening or enlarging of the ESG ducts. However, careful observation revealed the presence of numerous pan‐keratin‐positive cells surrounding the top of the ESG ducts that were unconnected, rather than forming cohesive outgrowths (Fig. 3G). This disorganization was in sharp contrast to young skin, in which pan‐keratin‐stained cells formed cohesive outgrowths above eccrine ducts (Fig. 3G). Taken together, these data suggest that reduced initial cell cohesion of ESG‐derived progeny contributes to reduced keratinocyte number and overall fewer cells in the repaired epidermis of aged vs. young wounded sites.

### Reduced cell cohesion in aging persists in repaired epidermis

**Figure 4:**
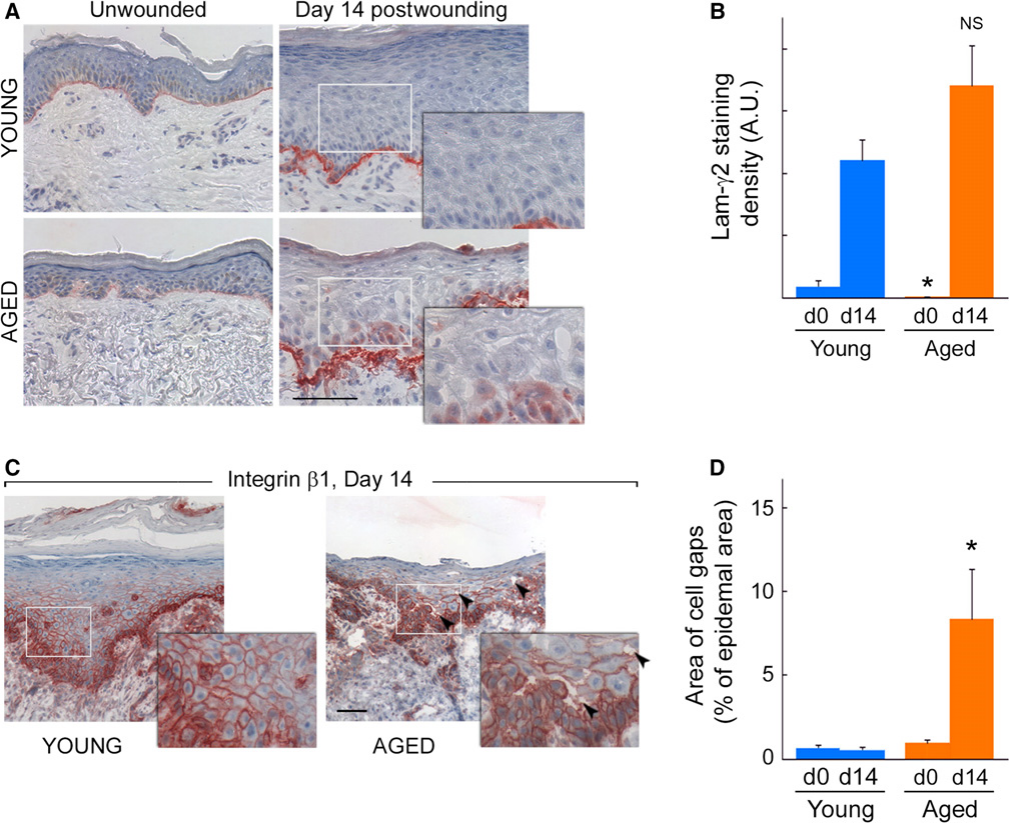
Reduced cell cohesion in aging persists in repaired epidermis. (A) Representative laminin‐γ2 immunostaining in young (top) and aged (bottom), unwounded (left) and 14 days post‐wounding (right) skin samples. Positive staining is red, and hematoxylin counterstaining is blue. While increased production of laminin‐γ2 is evident between wounded vs. unwounded skin, laminin γ2 levels between young and aged wounded skin are similar. However, deposition patterns are distinct between young and aged skin: laminin‐γ2 forms a thin layer beneath the repaired epidermis in young skin samples. In contrast, laminin‐γ2 spreads in the uneven contours of the damaged ECM. The resulting layer is relatively wider and more disorganized in aged vs. young repaired skin. Scale bar = 100 μm. Insets are magnified areas delineated by gray rectangles to show evidence of lack of epidermal cohesiveness in aged, but not young skin. Inset width = 142 μm. (B) Quantification of immunostaining presented in (A). *N* = 3/age group. *: *P* < 0.05 vs. young; NS = not statistically significant vs. young. (C) Beta1‐integrin staining of transversally cut skin sections obtained 14 days postwounding. Re‐epithelialization is complete, and repaired epidermis is differentiated in young (left) and aged (right) skin. Lack of epidermal cohesiveness is evident in aged (arrows) but not young skin. Scale bar = 100 μm; insets are magnified areas corresponding to gray rectangles. Inset width = 142 μm. (D) Quantification of extent of cell separation (‘area of cell gaps’) visible in A and C. *N* = 3/age group. *: *P* < 0.05 vs. young.

The migration of cells as a cohesive group, such as that observed in expanding outgrowths, is referred to as ‘collective cell migration’. Mechanical forces that develop at cell–cell junctions are directly influenced by the strength of interactions of cells with the extracellular matrix (ECM) (Discher *et al*., 2005; Maruthamuthu *et al*., 2011; Tseng *et al*., 2012; Checa *et al*., 2015). Because damage to the ECM (such as presence of fragmented collagen fibers, reduced ECM density, and decreased tissue mechanical forces) is a hallmark of aged skin [reviewed in (Rittié & Fisher, 2015)], we investigated keratinocyte–ECM interactions during outgrowth expansion in young and aged wounded skin. It was previously shown that laminin‐332 (formerly known as laminin‐5) mediates interaction between expanding keratinocytes and the dermal ECM in human wounds (Kainulainen *et al*., 1998; Nguyen *et al*., 2000). It was also suggested that new production of the γ2 chain of laminin‐332 is required for epidermal regeneration upon wounding (Kainulainen *et al*., 1998). Thus, we compared laminin‐γ2 expression in young and aged, normal and wounded skins as readout for keratinocyte/ECM interactions. As shown in Fig. 4A,B, we readily observed increased production of the γ2 chain of laminin in wounded vs. unwounded skin, both in young and aged individuals. Laminin γ2 levels were reduced in unwounded aged vs. young skin, but were not significantly different in wounded skin between the two age groups (Fig. 4B). However, we observed different deposition patterns between young and aged skin. While laminin γ2 was deposited in a well‐demarcated, thin layer beneath the repaired epidermis in young skin, laminin γ2 appeared much more diffuse and disorganized in aged repaired skin (Fig. 4A), following the uneven contours of the damaged ECM. These observations suggest differences in keratinocyte/ECM interactions between young and aged skins. Strikingly, we also observed gaps between keratinocytes within the repaired epidermis 2 weeks post‐wounding in aged skin (Fig. 4A, insets). Reduced cell cohesiveness in epidermis of aged vs. young skin was confirmed with β1‐integrin immunostaining, which highlights cell–cell contacts (Fig. 4C). Area of cell gaps was approximately eightfold higher in aged vs. young skins (Fig. 4D). In spite of this disorganization, the renewed epidermis showed stratification, change in keratinocyte orientation, and upper layers of granular keratinocytes (Fig. 4B,C). Altogether, these results indicate that altered keratinocyte/ECM interactions may contribute to persistent reduction in cell cohesion in wounded aged skin.

### Reduced cell cohesiveness in repaired epidermis of aged individuals is accompanied by reduced number of desmosomes

**Figure 5:**
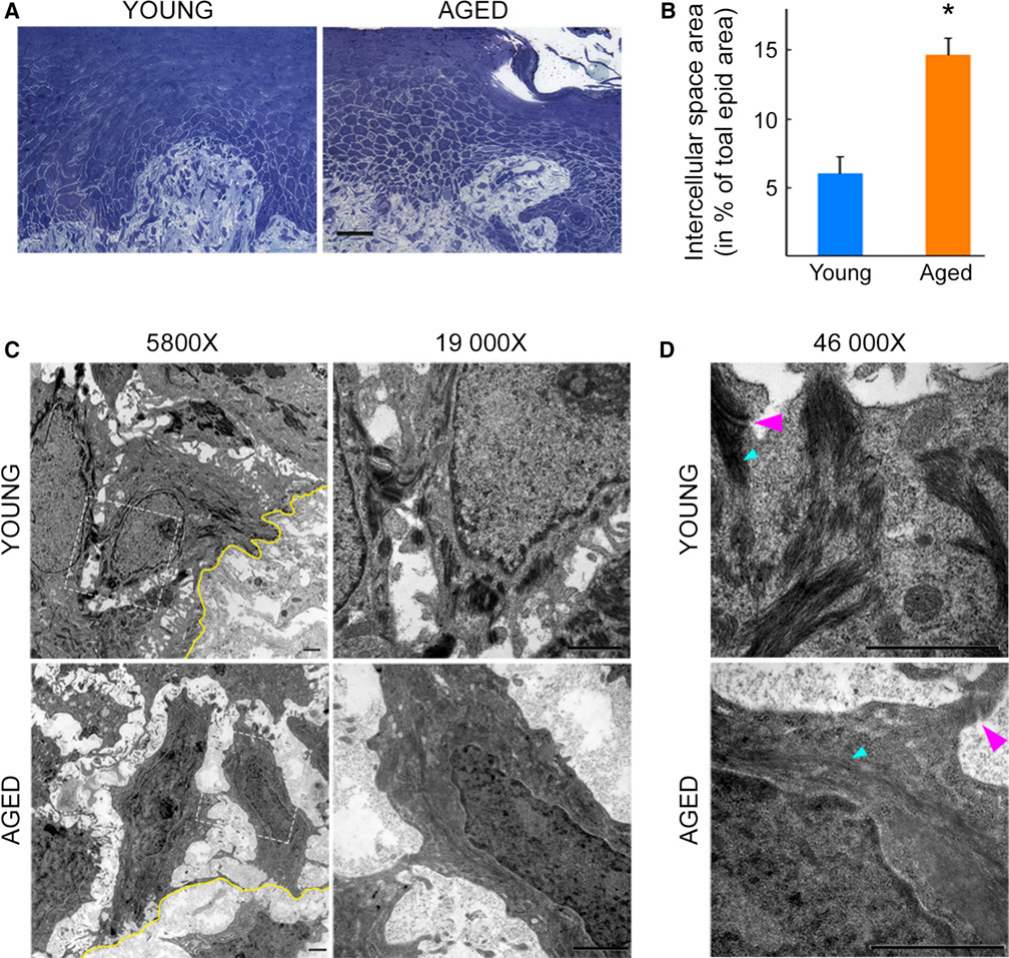
Reduced cell cohesiveness in repaired epidermis of aged individuals is accompanied by reduced number of desmosomes. (A) Toluidine blue staining of ultrathin skin sections from representative young (left) and aged (right) skin samples taken 14 days post‐wounding reveals greater intracellular spacing between basal and suprabasal keratinocytes in repaired epidermis of aged vs. young individuals. Scale bar = 50 μm. (B) Intracellular space area normalized to total epidermal area. Quantification was from toluidine blue‐stained samples taken 14 days post‐wounding. *N* = 3/age group. *: *P* < 0.05 vs. young. (C, D) Transmission electron microscopy images of skin samples taken 2 weeks post‐wounding from young (top) and aged (bottom) individuals. Scale bars = 1 μm. (C) 5800× magnification (left) reveals increased intracellular spacing and reduced number of cell–cell contacts in aged vs. young repaired epidermis. Yellow line delineates dermal–epidermal junction. Dashed squares demarcate magnified areas shown below at 19 000× magnification (right). Desmosomes are readily seen in young, but not aged skin. (D) 46 000× magnifications highlight mature desmosome (magenta arrowheads) connected with dense intermediate filaments (cyan arrowheads) in young skin (top). In contrast, desmosomes appear immature and keratin filaments are thin in aged repaired epidermis (bottom). Representative images, *N* = 3/age group.

To further investigate the reduced cohesion between keratinocytes in re‐epithelialized aged skin, we stained ultrathin sections prepared from epoxy resin‐embedded samples with toluidine blue. As shown in Fig. 5A, resin‐embedded samples yielded similar results, that is, greater intracellular spacing between keratinocytes in basal and suprabasal layers of repaired epidermis of aged vs. young skin. The difference in intracellular spacing between basal and suprabasal keratinocytes was significant, even when normalized to total epidermal area (14.6 ± 1.26% vs. 5.99 ± 1.22% in aged vs. young repaired epidermis, respectively, *n* = 3/age group, *P* = 0.0002, Fig. 5B). Interestingly, transmission electron microscopy not only also revealed increased intracellular spacing between keratinocytes in aged vs. young repaired epidermis, but also a reduced number of cell–cell contacts (Fig. 5B). Specifically, while desmosomes were readily seen in young repaired epidermis, they were much less abundant in repaired epidermis of older adults. When found, desmosomes appeared immature and connected to very thin intermediate filament bundles, in sharp contrast to mature desmosomes connected to dense keratin filaments in repaired epidermis of young skin (Fig. 5C). Taken together, these data further support the altered organization of repaired epidermis in elderly, compared with young skin. Reduced cell cohesiveness delays wound closure and persists in the repaired epidermis in aged skin.

## Discussion

The present study was designed to shed light on the cellular mechanisms for delayed wound re‐epithelialization in aging. We demonstrate that aging does not significantly alter the proliferation response in skin PSUs or ESGs upon wounding. We did not detect apoptotic keratinocytes in repaired epidermis of young or aged skin. Rather, aging is associated with reduced cohesive organization of keratinocytes forming the new epidermis. Reduced cell–cell cohesiveness was most obvious in ESG‐derived outgrowths that, when present, were often surrounded by unconnected cells in the scab overlaying aged wounds. We also demonstrate that reduced cell–cell cohesiveness persists during the repair process, with increased intercellular spacing, reduced number of desmosomes, and overall reduced number of keratinocytes in the repaired epidermis. Together, our results indicate that altered outgrowth formation (i) delays wound closure, (ii) reduces the efficiency of a robust wound‐induced proliferation response in elderly skin, and (iii) leads to a thinner repaired epidermis in aged vs. young skin.

A century ago, P. Lecomte du Noüy proposed an empirical formula to predict the velocity of repair and time of closure of a human aseptic wound (Du Noüy, 1916a). Interestingly, he described that the rate of *cicatrization* (French term for wound healing and scar tissue formation) is governed by two important factors: the size of the wound (Du Noüy, 1916a) and the age of the patient (Du Noüy, 1916b). Our measurements showing that healing of partial‐thickness wounds is delayed with aging is consistent with du Noüy's results.

Epidermal repair has been known to involve PSUs in rodents (Levy *et al*., 2007) and humans (Rittié *et al*., 2013). Here, we show that all cells in the outer layer of the sebaceous glands and the outer root sheath of the hair follicle (including the bulge) express proliferation marker for at least 5 days, both in young and aged skins (Fig. 2 and Rittié *et al*. (2013)). Our reconstructions of the few telogen hair follicles we saw suggest that the hair follicle response to wounding is independent of hair cycling. More samples would need to be examined to ascertain this observation. In contrast, we show that the cells of the arrector pili muscle, also part of the follicular structure (Poblet *et al*., 2004), do not increase their proliferation levels upon wounding. This observation suggests that muscle progenitors do not contribute to epithelial repair in skin. Cell proliferation in the bulge [where hair follicle stem cells reside (Ohyama *et al*., 2006; Rittié *et al*., 2009)] was reduced 7 days postwounding, whereas other areas remain hyperproliferative. Overall, these results are consistent with our previous report that aging does not significantly alter follicle density or bulge cell number in human scalp skin (Rittié *et al*., 2009). Thus, in addition to similar steady state number, our work suggests that human hair follicle stem cells retain their proliferative function with aging.

We have previously shown that ESGs are major contributors to re‐epithelialization of human skin wounds (Rittié *et al*., 2013), suggesting that human ESGs constitute an important reservoir of cells of high proliferative potential. Here, we precisely delineated that all cells of the outer layer of the absorption portion (coiled proximal duct and straight dermal duct) were positive for Ki67 during the re‐epithelialization process. We did not observe significant differences in proliferation between young and aged skins, nor did we observe a reduction in proliferation intensity in a subpart of the absorption portion (as we did for the bulge of hair follicles). Thus, it is unlikely that comparison of wound healing proliferative response between young and aged ESGs will be helpful in identifying a potential ESG stem cell niche. Based on label‐retaining properties and xenografts, Nakamura *et al*. reported that human ESG stem cells may reside in the coiled segment of the eccrine glands, that is, in the coiled duct and the secretory portion (Nakamura & Tokura, 2009). This conclusion contrasts with our current findings that wounding did not trigger a proliferation response in the secretory portion of sweat glands. In the absence of available stem cell markers, we were obviously not able to delineate the localization of the eccrine stem cell niche. However, our data suggest that the reservoir of cells of high proliferative potential may be located in the absorption portion of ESGs.

Whether aging affects the function of sweat glands in general remains controversial. Our results indicate that the proliferation response of ESGs to wounding is not altered *per se* with aging. Numerous reports indicate that aging alters thermoregulation function and heat tolerance in humans [reviewed in (Rittié & Fisher, 2015)]. Although the basis for this reduced physiological reaction remains unclear, local skin changes seem to be generally favored for underlying mechanisms, as opposed to sweat gland or central (hypothalamus) alterations (Inoue *et al*., 1999; Dufour & Candas, 2007; Hirata *et al*., 2012). Our results here are consistent with indirect effects of skin aging on ESG function.

Previous work from our group and others has characterized in detail the age‐associated damage to the skin dermal ECM, which includes increased collagen fiber fragmentation, reduced ECM resistance, and decreased tissue mechanical force [reviewed in (Rittié & Fisher, 2015)]. Although the ECM is well known for its role in providing structural scaffolds for embedded cells, recent studies have highlighted the importance of the ECM as underlying substrate for collective cell migration (Maruthamuthu *et al*., 2011; Tseng *et al*., 2012; Checa *et al*., 2015). For instance, increasing ECM rigidity (Young's modulus) enhances cellular traction forces and cell–cell adhesion (Discher *et al*., 2005; Maruthamuthu *et al*., 2011). Thus, it is likely that reduced rigidity of skin ECM, as it occurs with aging (Pailler‐Mattei *et al*., 2013), would reduce cell–cell cohesiveness as we observed *in vivo*. This reduced cell spreading was demonstrated *in vitro* with a collagen lattice model of ECM aging (Varani *et al*., 2009): When seeded on intact collagen, human epidermal keratinocytes rapidly attach and proliferate to cover the lattice overtime. When the collagen matrix was partially fragmented by matrix metalloproteinase‐1 prior to cell seeding (to mimic aged skin), keratinocytes attached, but surface coverage was reduced by half (Varani *et al*., 2009). Altogether, these observations suggest that damage to the ECM in aged skin may mediate reduced cell–cell cohesiveness and thereby reduce the efficiency of the re‐epithelialization process in aged skin. It is plausible that, because of the relatively smaller diameter of ESG ducts, ESG progeny are more susceptible to this phenomenon than PSU‐derived progeny (that could remain noticeable despite sub‐efficient outgrowth forming capacities). Conversely, because of relatively larger diameter, PSU‐derived cells might not sense the effects of ECM alterations to the same extent as the smaller ESG‐derived outgrowths. Additional research will be required to identify the molecular basis and regional details of altered cell–cell and cell–ECM adhesion.

**Figure 6:**
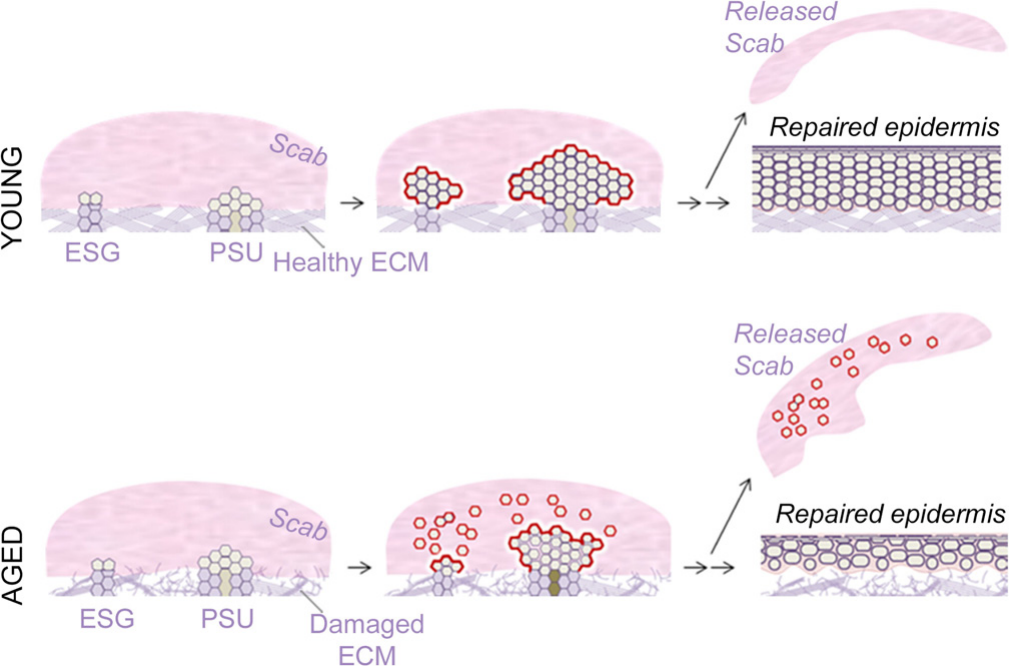
Hypothetical scheme for inefficient re‐epithelialization process in aged skin. In young individuals (top), wounding triggers a strong proliferation response in eccrine sweat glands (ESGs) and pilo‐sebaceous units (PSUs). *Top (young)*: ESG and PSU progeny form cohesive outgrowths that expand between the scab and healthy extracellular matrix (ECM). Upon optimal collective growth and migration, the proliferation phase of wounding healing gives rise to a thick, hyperplastic epidermis. *Bottom (aged)*: wounding also triggers a strong proliferation response in ESGs and PSUs. However, reduced cell–cell contacts between ESG and PSU progeny lessen outgrowth cohesion and outgrowth cell number, thereby slowing wound closure. In addition, the resulting repaired epidermis is much thinner than that of young individuals.

Our proposed scheme for inefficient re‐epithelialization in aged skin is summarized in Fig. 6 and as follows: In the skin of young adults, partial‐thickness wounding triggers a robust proliferation response in ESGs and PSUs that generates cohesive keratinocyte outgrowths that expand overtime. Re‐epithelialization is complete when outgrowths merge with each other and the scab falls off the surface of the wounded area. The end result is a hyperplastic, repaired epidermis that will further undergo remodeling. In the skin of aged individuals, partial‐thickness wounding triggers a similarly robust proliferation response in ESGs and PSUs. However, the damaged ECM of aged skin is a poor substrate for cohesive migration of the keratinocyte progeny. As a result, outgrowths (especially the smaller ESG‐derived outgrowths) do not form as compact cell stacks and many disconnected cells get trapped in the scab. The few, smaller outgrowths that are formed still merge with each other, but the completion of re‐epithelialization is delayed. Many of the original cells fall off with the scab and the resulting repaired epidermis is relatively thin and less cohesive compared with young skin.

In conclusion, our study provides new insights into the cellular mechanism of delayed wound healing in the elderly. In fact, healing is not delayed; rather, it is slowed due to reduced outgrowths from ESGs. By demonstrating that reduced ESG outgrowth results from impairment of keratinocyte cohesive organization, likely mediated by age‐related damage to the underlying ECM, our results highlight the critical importance of the wound environment.

## Experimental procedures

### Research subject recruitment, treatment, and tissue procurement

All protocols were approved by the Institutional Review Board of the University of Michigan, and each human subject provided written informed consent prior to entering the study. Human subjects’ recruitment and treatment was performed as previously described (Rittié *et al*., 2011, 2013) with slight modifications. Briefly, partial‐thickness wounds were made on 1% lidocaine‐anesthetized discrete areas of the dorsal surface of the forearm using three passes of a fractional CO_2_ laser (UltraPulse Encore, Lumenis, San Jose, CA, USA) used in continuous wave mode and set at 100 mJ, 60 W, with settings of 3/5/6. Generated partial‐thickness wounds were similar to those obtained previously with the Ultrapulse laser (Rittié *et al*., 2011, 2013). The ~ 5‐mm^2^ CO_2_ laser‐generated wounds were gently rinsed with tap water and covered with semipermeable dressing (Tegaderm, 3M, Minneapolis, MN, USA). Full‐thickness punch biopsies (4‐mm) were taken under local anesthesia (1% lidocaine) from the center of wounds at various time points after laser treatment. Skin samples were embedded in Tissue‐Tek OCT compound (Miles Scientific Labs, Naperville, IL, USA), frozen in liquid nitrogen, and stored at −80 °C until processing.

A total of 18 subjects were enrolled in the ‘young’ group (8 men, 10 women; 25 to 40 years of age —mean: 31.5, median: 31.0), and 18 subjects were enrolled in the ‘aged’ group (8 men, 10 women; 70 to 84 years of age—mean: 77.3, median: 78.5).

### Immunohistochemistry and imaging

Immunohistochemistry (IHC) was performed on 7‐, 10‐, or 20‐μm‐thick frozen skin sections fixed in 2% paraformaldehyde or ice‐cold acetone, using the following primary antibodies: anti‐pan‐keratin antibodies were as previously described (Rittié *et al*., 2013); anti‐Ki67 was from Biogenex (Fremont, CA, USA); anti‐β1‐integrin was from EMD Millipore (Temecula, CA, USA); and anti‐laminin‐γ2 was from AbCam (Cambridge, MA, USA). IHC was performed as previously described (Rittié *et al*., 2008), and slides were mounted with 90% glycerin or ProLong Gold medium (both from Thermo Fisher Scientific, Waltham, MA, USA). Imaging of horizontally cut sections was performed with a Leica MXFL III Stereo Microscope (Leica Microsystems, Buffalo Grove, IL, USA; accessed at the Microscopy and Image‐Analysis Laboratory, Biomedical Research Core Facilities, University of Michigan Medical School). Other imaging was performed with an Axioscope 2 (Carl Zeiss, Thornwood, NY, USA) equipped with a Spot digital camera (Spot Imaging Solutions, Sterling Heights, MI, USA). Large areas were captured on multiple overlapping sections, and images were merged using Photostitch 3.1 software (Canon, Lake Success, NY, USA). A microscope micrometer (Thermo Fisher Scientific) was used during imaging for image calibration.

### Computer‐assisted 3D reconstructions

A series of ~ 150–200 14‐ to 20‐μm‐thick horizontally cut, consecutive, immunostained sections was generated for each biopsy. Digital images were imported in reconstruct 1.1 software (Fiala, 2005) and calibrated. Reconstruction was performed as previously described in detail [see supplementary information in (Rittié *et al*., 2013)]. Still and animated 3D renderings were generated using blender 2.68 (http://www.blender.org/development/release-logs/blender-268). Scale bars (500 μm‐long) were generated in reconstruct, imported in Blender with corresponding 3D model, and positioned on the forwardmost plan of the front hair follicle. Supplemental videos are supplied as .mov files containing H.264‐encoded data.

### Quantification methods

Area of new epidermis was measured on calibrated still, top view 3D images with reconstruct 1.1. Percent re‐epithelialization = epidermis area/total biopsy area × 100. Epidermal thickness was not taken into account in these calculations. Epidermal volumes were extracted from calibrated traces in reconstruct.

Appendage (i.e., PSU and ESG) count was made on still or animated 3D renderings. Percent contributing ESGs to wound closure = number of ESGs topped with an outgrowth/number of total ESGs × 100. Similar equation was used for PSUs. For each sample, all outgrowths that appeared shared between an ESG and a PSU (i.e., was of uncertain origin) were not included in calculation. When several biopsies were available for an individual (e.g., multiple time points), results were grouped.

Ki67 staining density was calculated from raw data generated in reconstruct, dividing the Ki67 count by the total volume of the respective appendage subunit (ESG ducts and coils, hair follicles, sebaceous glands, and arrector pili muscles). For ESGs, ‘ducts’ designates the descending portion of the gland until the first elbow. Each average was calculated from subunits of 11 young and 14 aged ESGs, and 9 young and 5 aged PSUs from 2 to 3 subjects per age group, except for aged arrector pili (*n* = 3).

Laminin‐γ2 IHC staining density was measured with imagej 1.49v (http://imagej.nih.gov) on the isolated red staining channel, using percent area from pixel counts of the thresholded image. Staining outside the dermal–epidermal area, if any (e.g., around appendages) was excluded. Four‐to‐six 400× images encompassing the width of the biopsy were analyzed for each subject. Measurements were averaged among three subjects per age group.

Extent of cell separation (‘area of cell gaps’) was measured with imagej and represents areas of background color within the epidermis. Pixel counts were normalized to the total surface of the epidermis (area between dermal–epidermal junction and top of granular layer (stratum corneum excluded)). Three images each of random but not overlapping location across the width of the biopsy were analyzed for each subject. Measurements were averaged among three subjects per age group.

Surface area occupied by individual cells was measured with IMAGEJ on β1‐integrin IHC‐calibrated digital images (400× magnification). A minimum of 10 basal keratinocytes was evaluated for each image, and measurements were averaged among 6 subjects per age group.

Number of basal keratinocytes was evaluated on β1‐integrin or laminin‐γ2 IHC‐calibrated digital images with imagej (cell counter plugin, http://imagej.nih.gov/ij/plugins/cell-counter.html) and normalized to length of dermal–epidermal junction (also measured with imagej). The entire width of 2 randomly taken 400× images was used for each subject, and measurements were averaged among 6 subjects per age group.

Epidermal thickness was evaluated on histology or IHC‐calibrated digital images with imagej: length between dermal–epidermal junction and top of granular layer (stratum corneum excluded) was measured roughly every 100–150 μm, on the entire width of 2–4 randomly chosen 100× images for each subject, for an average of 35 measurements per time point. Values for each time points were averaged among 7–9 subjects per age group.

### Apoptosis detection

Apoptosis was detected *via* DNA fragmentation by the TUNEL assay with the ‘ApopTag^®^ Red *In Situ* Apoptosis Detection’ kit (EMD Millipore) following the manufacturer's instructions. Positive control was obtained by digesting unwounded skin samples with 2.5 U DNase I (Sigma, Saint‐Louis, MO, USA) for 10 min at room temperature. Positive fluorescent staining was pseudocolored in red and DAPI nuclear counter staining in blue. Blue and red signals were separated from skin autofluorescence signal with the spectral unmixing plugin of imagej.

### Plastic embedding for toluidine blue staining and Transmission Electron Microscopy

Four‐millimeter full‐thickness skin biopsies were fixed overnight in 2% (v/v) glutaraldehyde in 0.1 mm cacodylate buffer, pH 7.4 (Sigma), rinsed in Sorensen buffer (1 mm KH_2_PO_4_, 3 mm Na_2_HPO_4_, pH 7.4), postfixed 1.5 h in 1% osmium tetroxide, rinsed in Sorensen buffer, and stored at 4 °C until processing. Skin samples were dehydrated with graded ethanol followed by propylene oxide (EM Sciences), embedded in EPON^™^ epoxy resin, and sectioned at 900 nm thickness. Ultrathin sections were either stained with toluidine blue and mounted in Permount (Thermo Fisher Scientific) for light microscopy, or transferred onto a grid for transmission electron microscopy (performed on a Phillips CM‐100 (Eindhoven, The Netherlands), accessed at the Microscopy and Image‐Analysis Laboratory core facility).

### Charts and statistics

Charts were generated with Microsoft Excel 2010 or graphpad prism 6.0 (La Jolla, CA) and assembled in Photoshop CC. Bar graphs represent means ± SEM. Statistical analyses were performed with graphpad prism 6.0. Comparisons among groups were made with the Wilcoxon signed rank test, the Holm multiple comparison test, or multiple comparison after one‐way ANOVA test. All *P* values are 2‐tailed, and significance was defined as *P* < 0.05 (depicted by asterisks on figures). Non‐significant differences were indicated with ‘NS’.

## Author contributions

LR and GJF conceived experiments and secured funding. LR, EAF, and JSO performed experiments. LR and EAF analyzed data. LR and GJF interpreted the results. LR wrote the manuscript, and GJF revised it critically for important intellectual content. JSO and JJV provided expertise and feedback. All authors revised the manuscript.

## Funding

This research was supported by the University of Michigan Dermatology Department research fund, NIH K01 grant AR059678 (to LR), and grant from the University of Michigan Nathan Shock Center Functional Assessment Core supported by NIH AG013283 (to LR). The Microscopy and Image‐analysis Laboratory is a multi‐user imaging facility supported by the University of Michigan.

## Conflict of interest

None declared.

## Supporting information


**Video S1** Animated version for Fig.  1A–D (H.264 encoding). Revolving reconstructions of wounded skin samples of young and aged individuals taken 5 and 7 days post‐wounding (starting at 1 s or frame 1 and 20 s or frame 415, respectively), as labeled in the video.

**Video S2** Animated version for Fig. 2A–C (H.264 encoding). Revolving reconstructions of PSU and ESG stained with Ki67. The video shows, successively, i) (starts at 1 s, or frame 1) anagen PSU from aged individual 5 days post‐wounding; scale bar (500 μm‐long) was positioned at the forwardmost plan of the taller hair follicle, ii) (starts at 25 s, or frame 503) telogen PSU and ESG from young individual 7 days post‐wounding, and iii) (starts at 53 s, or frame 1067) ESG from aged individual 5 days post‐wounding, as labeled in the video.

## References

[acel12493-bib-0001] Allman RM (1997) Pressure ulcer prevalence, incidence, risk factors, and impact. Clin. Geriatr. Med. 13, 421–436. 9227937

[acel12493-bib-0002] Arya AK , Tripathi R , Kumar S , Tripathi K (2014) Recent advances on the association of apoptosis in chronic non healing diabetic wound. World J. Diabetes 5, 756–762. 25512778 10.4239/wjd.v5.i6.756 PMC4265862

[acel12493-bib-0003] Bruno S , Darzynkiewicz Z (1992) Cell cycle dependent expression and stability of the nuclear protein detected by Ki‐67 antibody in HL‐60 cells. Cell Prolif. 25, 31–40. 1540682 10.1111/j.1365-2184.1992.tb01435.x

[acel12493-bib-0004] Checa S , Rausch MK , Petersen A , Kuhl E , Duda GN (2015) The emergence of extracellular matrix mechanics and cell traction forces as important regulators of cellular self‐organization. Biomech. Model. Mechanobiol. 14, 1–13. 24718853 10.1007/s10237-014-0581-9

[acel12493-bib-0005] Desmoulière A , Redard M , Darby I , Gabbiani G (1995) Apoptosis mediates the decrease in cellularity during the transition between granulation tissue and scar. Am. J. Pathol. 146, 56–66. 7856739 PMC1870783

[acel12493-bib-0006] Discher DE , Janmey P , Wang YL (2005) Tissue cells feel and respond to the stiffness of their substrate. Science 310, 1139–1143. 16293750 10.1126/science.1116995

[acel12493-bib-0007] Du Noüy PL (1916a) Cicatrization of wounds: II. Mathematical expression of the curve representing cicatrization. J. Exp. Med. 24, 451–460. 19868053 10.1084/jem.24.5.451 PMC2125479

[acel12493-bib-0008] Du Noüy PL (1916b) Cicatrization of wounds: III. The relation between the age of the patient, the area of the wound, and the index of cicatrization. J. Exp. Med. 24, 461–470. 19868054 10.1084/jem.24.5.461 PMC2125475

[acel12493-bib-0009] Dufour A , Candas V (2007) Ageing and thermal responses during passive heat exposure: sweating and sensory aspects. Eur. J. Appl. Physiol. 100, 19–26. 17242944 10.1007/s00421-007-0396-9

[acel12493-bib-0010] Eaglstein WH (1989) Wound healing and aging. Clin. Geriatr. Med. 5, 183–188. 2645996

[acel12493-bib-0011] Ernst N , Yay A , Biro T , Tiede S , Humphries M , Paus R , Kloepper JE (2013) β1 integrin signaling maintains human epithelial progenitor cell survival in situ and controls proliferation, apoptosis and migration of their progeny. PLoS One 8, e84356. 24386370 10.1371/journal.pone.0084356 PMC3874009

[acel12493-bib-0012] Fiala JC (2005) Reconstruct: a free editor for serial section microscopy. J. Microsc. 218, 52–61. 15817063 10.1111/j.1365-2818.2005.01466.x

[acel12493-bib-0013] Gavrieli Y , Sherman Y , Ben‐Sasson SA (1992) Identification of programmed cell death in situ via specific labeling of nuclear DNA fragmentation. J. Cell Biol. 119, 493–501. 1400587 10.1083/jcb.119.3.493 PMC2289665

[acel12493-bib-0014] Gerstein AD , Phillips TJ , Rogers GS , Gilchrest BA (1993) Wound healing and aging. Dermatol. Clin. 11, 749–757. 8222358

[acel12493-bib-0015] Gosain A , DiPietro LA (2004) Aging and wound healing. World J. Surg. 28, 321–326. 14961191 10.1007/s00268-003-7397-6

[acel12493-bib-0016] Grose R , Hutter C , Bloch W , Thorey I , Watt FM , Fassler R , Brakebusch C , Werner S (2002) A crucial role of beta 1 integrins for keratinocyte migration in vitro and during cutaneous wound repair. Development 129, 2303–2315. 11959837 10.1242/dev.129.9.2303

[acel12493-bib-0017] Hirata A , Nomura T , Laakso I (2012) Computational estimation of decline in sweating in the elderly from measured body temperatures and sweating for passive heat exposure. Physiol. Meas. 33, N51–N60. 22814101 10.1088/0967-3334/33/8/N51

[acel12493-bib-0018] Holt DR , Kirk SJ , Regan MC , Hurson M , Lindblad WJ , Barbul A (1992) Effect of age on wound healing in healthy human beings. Surgery 112, 293–297. 1641768

[acel12493-bib-0019] Inoue Y , Shibasaki M , Ueda H , Ishizashi H (1999) Mechanisms underlying the age‐related decrement in the human sweating response. Eur. J. Appl. Physiol. 79, 121–126. 10.1007/s004210050485 10029332

[acel12493-bib-0020] Jaul E (2009) Non‐healing wounds: the geriatric approach. Arch. Gerontol. Geriatr. 49, 224–226. 18838182 10.1016/j.archger.2008.08.005

[acel12493-bib-0021] Kainulainen T , Hakkinen L , Hamidi S , Larjava K , Kallioinen M , Peltonen J , Salo T , Larjava H , Oikarinen A (1998) Laminin‐5 expression is independent of the injury and the microenvironment during reepithelialization of wounds. J. Histochem. Cytochem. 46, 353–360. 9487117 10.1177/002215549804600309

[acel12493-bib-0022] Kim DJ , Mustoe T , Clark RA (2015) Cutaneous wound healing in aging small mammals: a systematic review. Wound Repair Regen. 23, 318–339. 25817246 10.1111/wrr.12290

[acel12493-bib-0023] Levy V , Lindon C , Zheng Y , Harfe BD , Morgan BA (2007) Epidermal stem cells arise from the hair follicle after wounding. FASEB J. 21, 1358–1366. 17255473 10.1096/fj.06-6926com

[acel12493-bib-0024] Lobitz WC Jr , Holyoke JB , Montagna W (1954) Responses of the human eccrine sweat duct to controlled injury: growth center of the epidermal sweat duct unit. J. Invest. Dermatol. 23, 329–344. 13212169 10.1038/jid.1954.116

[acel12493-bib-0025] Lopez‐Rovira T , Silva‐Vargas V , Watt FM (2005) Different consequences of beta1 integrin deletion in neonatal and adult mouse epidermis reveal a context‐dependent role of integrins in regulating proliferation, differentiation, and intercellular communication. J. Invest. Dermatol. 125, 1215–1227. 16354192 10.1111/j.0022-202X.2005.23956.x

[acel12493-bib-0026] Marples MJ (1965) The Ecology of the Human Skin. Springfield, IL: Charles C. Thomas.

[acel12493-bib-0027] Maruthamuthu V , Sabass B , Schwarz US , Gardel ML (2011) Cell‐ECM traction force modulates endogenous tension at cell‐cell contacts. Proc. Natl Acad. Sci. U.S.A. 108, 4708–4713. 21383129 10.1073/pnas.1011123108 PMC3064395

[acel12493-bib-0028] Nakamura M , Tokura Y (2009) The localization of label‐retaining cells in eccrine glands. J. Invest. Dermatol. 129, 2077–2078. 19177140 10.1038/jid.2008.443

[acel12493-bib-0029] Nguyen BP , Ryan MC , Gil SG , Carter WG (2000) Deposition of laminin 5 in epidermal wounds regulates integrin signaling and adhesion. Curr. Opin. Cell Biol. 12, 554–562. 10978889 10.1016/s0955-0674(00)00131-9

[acel12493-bib-0030] Ohyama M , Terunuma A , Tock CL , Radonovich MF , Pise‐Masison CA , Hopping SB , Brady JN , Udey MC , Vogel JC (2006) Characterization and isolation of stem cell‐enriched human hair follicle bulge cells. J. Clin. Invest. 116, 249–260. 16395407 10.1172/JCI26043 PMC1323261

[acel12493-bib-0031] Orringer JS , Kang S , Johnson TM , Karimipour DJ , Hamilton T , Hammerberg C , Voorhees JJ , Fisher GJ (2004) Connective tissue remodeling induced by carbon dioxide laser resurfacing of photodamaged human skin. Arch. Dermatol. 140, 1326–1332. 15545540 10.1001/archderm.140.11.1326

[acel12493-bib-0032] Pailler‐Mattei C , Debret R , Vargiolu R , Sommer P , Zahouani H (2013) In vivo skin biophysical behaviour and surface topography as a function of ageing. J. Mech. Behav. Biomed. Mater. 28, 474–483. 23664827 10.1016/j.jmbbm.2013.04.008

[acel12493-bib-0033] Poblet E , Jimenez F , Ortega F (2004) The contribution of the arrector pili muscle and sebaceous glands to the follicular unit structure. J. Am. Acad. Dermatol. 51, 217–222. 15280840 10.1016/j.jaad.2004.01.054

[acel12493-bib-0034] Rittié L , Fisher GJ (2015) Natural and sun‐induced aging of human skin. Cold Spring Harb. Perspect Med. 5, a015370. 25561721 10.1101/cshperspect.a015370 PMC4292080

[acel12493-bib-0035] Rittié L , Kang S , Voorhees JJ , Fisher GJ (2008) Induction of collagen by estradiol: difference between sun‐protected and photodamaged human skin in vivo. Arch. Dermatol. 144, 1129–1140. 18794456 10.1001/archderm.144.9.1129

[acel12493-bib-0036] Rittié L , Stoll SW , Kang S , Voorhees JJ , Fisher GJ (2009) Hedgehog signaling maintains hair follicle stem cell phenotype in young and aged human skin. Aging Cell 8, 738–751. 20050020 10.1111/j.1474-9726.2009.00526.x

[acel12493-bib-0037] Rittié L , Perbal B , Castellot JJ Jr , Orringer JS , Voorhees JJ , Fisher GJ (2011) Spatial‐temporal modulation of CCN proteins during wound healing in human skin in vivo. J. Cell Commun. Signal. 5, 69–80. 21484592 10.1007/s12079-010-0114-y PMC3058195

[acel12493-bib-0038] Rittié L , Sachs DL , Orringer JS , Voorhees JJ , Fisher GJ (2013) Eccrine sweat glands are major contributors to reepithelialization of human wounds. Am. J. Pathol. 182, 163–171. 23159944 10.1016/j.ajpath.2012.09.019 PMC3538027

[acel12493-bib-0039] Sato K , Dobson RL (1970) Regional and individual variations in the function of the human eccrine sweat gland. J. Invest. Dermatol. 54, 443–449. 5446389 10.1111/1523-1747.ep12259272

[acel12493-bib-0040] Sen CK , Gordillo GM , Roy S , Kirsner R , Lambert L , Hunt TK , Gottrup F , Gurtner GC , Longaker MT (2009) Human skin wounds: a major and snowballing threat to public health and the economy. Wound Repair Regen. 17, 763–771. 19903300 10.1111/j.1524-475X.2009.00543.x PMC2810192

[acel12493-bib-0041] Sgonc R , Gruber J (2013) Age‐related aspects of cutaneous wound healing: a mini‐review. Gerontology 59, 159–164. 23108154 10.1159/000342344

[acel12493-bib-0042] Thomas DR (2001) Age‐related changes in wound healing. Drugs Aging 18, 607–620. 11587247 10.2165/00002512-200118080-00005

[acel12493-bib-0043] Tseng Q , Duchemin‐Pelletier E , Deshiere A , Balland M , Guillou H , Filhol O , Thery M (2012) Spatial organization of the extracellular matrix regulates cell‐cell junction positioning. Proc. Natl Acad. Sci. U.S.A. 109, 1506–1511. 22307605 10.1073/pnas.1106377109 PMC3277177

[acel12493-bib-0044] Varani J , Perone P , Deming MO , Warner RL , Aslam MN , Bhagavathula N , Dame MK , Voorhees JJ (2009) Impaired keratinocyte function on matrix metalloproteinase‐1 (MMP‐1) damaged collagen. Arch. Dermatol. Res. 301, 497–506. 19352688 10.1007/s00403-009-0948-4 PMC2908395

[acel12493-bib-0045] Weller R , Pattullo S , Smith L , Golden M , Ormerod A , Benjamin N (1996) Nitric oxide is generated on the skin surface by reduction of sweat nitrate. J. Invest. Dermatol. 107, 327–331. 8751965 10.1111/1523-1747.ep12363167

